# Integrated Genomics and Transcriptomics Provide Insights into Salt Stress Response in *Bacillus subtilis* ACP81 from Moso Bamboo Shoot (*Phyllostachys praecox*) Processing Waste

**DOI:** 10.3390/microorganisms12020285

**Published:** 2024-01-29

**Authors:** Qiaoling Li, Zhiyuan Huang, Zheke Zhong, Fangyuan Bian, Xiaoping Zhang

**Affiliations:** 1 China National Bamboo Research Center, Key Laboratory of State Forestry and Grassland Administration on Bamboo Forest Ecology and Resource Utilization, Hangzhou 310012, China; qiaolingli@caf.ac.cn (Q.L.); zhiyuanhuang@caf.ac.cn (Z.H.); zhekez@163.com (Z.Z.); bianfangyuan@caf.ac.cn (F.B.); 2 National Long-Term Observation and Research Station for Forest Ecosystem in Hangzhou-Jiaxing-Huzhou Plain, Hangzhou 310012, China; 3 Engineering Research Center of Biochar of Zhejiang Province, Hangzhou 310012, China

**Keywords:** *Bacillus subtilis*, salt tolerance, osmoregulatory substances, flagellar motility, phosphotransferase system

## Abstract

Salt stress is detrimental to the survival of microorganisms, and only a few bacterial species produce hydrolytic enzymes. In this study, we investigated the expression of salt stress-related genes in the salt-tolerant bacterial strain *Bacillus subtilis* ACP81, isolated from bamboo shoot processing waste, at the transcription level. The results indicate that the strain could grow in 20% NaCl, and the sub-lethal concentration was 6% NaCl. Less neutral protease and higher cellulase and β-amylase activities were observed for *B. subtilis* ACP81 under sub-lethal concentrations than under the control concentration (0% NaCl). Transcriptome analysis showed that the strain adapted to high-salt conditions by upregulating the expression of genes involved in cellular processes (membrane synthesis) and defense systems (flagellar assembly, compatible solute transport, glucose metabolism, and the phosphotransferase system). Interestingly, genes encoding cellulase and β-amylase-related (malL, celB, and celC) were significantly upregulated and were involved in starch and sucrose metabolic pathways, and the accumulated glucose was effective in mitigating salt stress. RT-qPCR was performed to confirm the sequencing data. This study emphasizes that, under salt stress conditions, ACP81 exhibits enhanced cellulase and β-amylase activities, providing an important germplasm resource for saline soil reclamation and enzyme development.

## 1. Introduction

Soil salinity is a major factor affecting crop yield, and it has been predicted that half of the world’s agricultural land will be saline, and that humanity will face global food shortages by 2050 [1,2]. China ranks third globally in terms of the size of salinized land area. There are various methods for making use of salinized lands. One of them is land reclamation, which can be used to reclaim previous agricultural lands to alleviate the problem of arable land loss [3]. Moreover, biological improvement measures are regarded as an effective way to combat soil salinization. A common method is enabling plants to adapt or even exploit soil salt stress using bacteria which secrete metabolites to promote plant growth via their cellular structures (flagella) or biological metabolic processes [4]. Bacterial cell membrane proteins (signal transduction proteins, recognition receptors, ion transport proteins, structural proteins, and enzymes) play important roles in selective uptake and waste discharge [5]. Additionally, abiotic and biotic stresses in plants are minimized when salt-tolerant bacteria colonize their internal tissues [6,7]. Therefore, salt-tolerant bacteria play an important role in soil remediation and plant survival in saline soils.

*Bacillus subtilis* has certain advantages in saline–alkali land improvement by inhibiting the accumulation of excessive sodium, enhancing the activities of soil enzyme (including urease, protease, invertase, and catalase), and decreasing oxidative damage, thereby improving nutrient uptake by crops [8,9]. Moreover, *B. subtilis* has a high proliferation rate and releases a wide range of active substances that can protect plants from diseases and increase plant yield [10]. Studies found that highly active *B. subtilis* can increase the yield of wheat and cotton grown in extremely saline soils by 18% and 65.52%, respectively [9,11]. Furthermore, select strains of *Bacillus* spp. have been used in farming and food wastewater applications [3,12]. This approach not only relieved the pressure of eutrophication in water bodies and reduced the cost of water treatment but also improved soil fertility. The waste generated during the production of bamboo shoots is rich in dietary fiber, soluble sugars, and other nutrients that can serve as source of carbon, energy, and trace elements for bacteria [13]. Nevertheless, there are few studies on the salt tolerance mechanisms of *B. subtilis* and information on the response of its hydrolase activity to salt stress is lacking.

Bacteria that produce cellulase and amylase are rare, and their enzyme activities are directly affected by environmental conditions. *Bacillus* spp. have been used to manufacture industrial enzymes, including amylase, glycosyltransferases, cellulase, and penicillinase [14,15]. However, it remains unclear whether the expression of the genes encoding cellulase and β-amylase in *B. subtilis* is affected by salt stress conditions and whether this has important implications for gaining insight into its cellulase regulatory mechanisms and applications.

In this study, the salt-tolerant bacterial strain *B. subtilis* ACP81 was isolated from bamboo shoot-processing waste. We further tested the effect of a sub-lethal salt concentration on the hydrolytic enzyme activities of *B. subtilis* ACP81. Moreover, the complete genome, transcriptome, and morphology of *B. subtilis* ACP81 in response to sub-lethal salt concentrations were assessed to reveal the genetic mechanism underlying the salt tolerance of this strain. This study is the first to isolate a salt-tolerant strain from bamboo shoot processing waste and suggests the strain as a resource for enzyme production or soil remediation.

## 2. Materials and Methods

### 2.1. Isolation and Identification of Bacteria

Bamboo shoot waste samples were collected from a Moso bamboo plantation in Anji County, Zhejiang Province, China. The bamboo shoot-processing waste was diluted 100- and 1000-fold, spread on functional medium, and incubated at 28 °C for 24 to 48 h. The target strain was isolated by the pour plate and gradient dilution method using lysogeny broth, skim milk agar medium, soluble starch medium, and sodium carboxymethylcellulose medium. Based on the clear water circles zones and chromogenic reactions on the functional medium, colonies with the highest ability to hydrolyze protein, starch, and cellulose hydrolysis were selected and grown. The strain with the largest clear zone, named ACP81, was subjected to DNA extraction using a genomic DNA purification kit (TSP101–200; Tsingke Biotech, Beijing, China). Universal primers 27 F (5’–AGTTTGATCMTGGCTCAG–3’) and 1492R (5’–GGTTACCTTGTTACGACTT–3’) were used to amplify bacterial 16S rRNA genes, and a LongGene A300 DNA analyzer was used to perform sequencing. A phylogenetic tree was constructed by comparing the 16S rRNA sequences of the target outlier strains with those of closely related type strains in the NCBI GenBank (BLAST score of 99%).

### 2.2. Effect of Salt Stress on the Growth, Hydrolytic Enzymes, and Antioxidant Enzyme of Bacillus subtilis ACP81

For the salt stress test, the selected strains were inoculated in lysogeny broth medium containing different concentrations of NaCl (0, 2, 6, 11, 16, and 20% *w*/*w*). Growth was measured at OD600 using a fully automated bacterial growth curve meter (Bioscreen C Pro, Helsinki, Finland). Based on the growth curves of the target strain in salt-stressed environments, the sub-lethal concentration was determined for subsequent experiments.

*B. Subtilis* ACP81 cells were collected after cultivation in LB broth containing either 0% or 6% NaCl for 12 h. The bacterial solution was centrifuged (10,000× *g*, 4 °C) for 10 min, washed twice with sterilized water, and resuspended in sterilized water. The bacterial suspension was sonicated on ice (20 kHz, 15 min), centrifuged (10,000 rpm, 4 °C) for 5 min, and the supernatant retained as a crude enzyme extract. Protein concentration of the extracts was determined with the BCA Protein Assay Kit (Cominbio, Suzhou, China) using bovine serum albumin as standard. The activities of the hydrolytic enzymes cellulase (CL), β-amylase, and neutral protease (NP), the antioxidant enzymes superoxide dismutase (SOD), catalase (CAT), and peroxidase (POD), and the levels of malondialdehyde (MDA) and oxygen free radicals (OFR) were determined using enzyme kits (Cominbio). All experiments were performed in triplicate.

The morphological structure of the *B. subtilis* ACP81 under salt stress conditions (sub-lethal concentrations) was observed by transmission electron microscopy (TEM) using a Regulus 8100 instrument (Hitachi High-Tech Corp., Tokyo, Japan).

### 2.3. Genomic DNA and Whole-Transcriptome Sequencing

The Wizard^®^ Genomic DNA Purification Kits (Promega, Madison, WI, USA) were used to extract bacterial DNA from the ACP81 isolate. Purified genomic DNA was quantified using the TBS-380 fluorometer (Turner BioSystems Inc., Sunnyvale, CA, USA). High-quality DNA (OD260/280 = 1.8–2.0) was sequenced using an Illumina (San Diego, CA, USA) sequencing platform.

Cell samples of *B. subtilis* ACP81 were collected during the exponential phase at no salt (0% NaCl) and sub-lethal growth dosed (6% NaCl). The transcriptome analysis was performed using three biological replicates. Total RNA was extracted from *B. subtilis* ACP81 cultured at various salt concentrations using the TRIzol method (Invitrogen, Shanghai, China). The RNA quality was determined by 2100 Bioanalyser (Agilent, Santa Clara, CA, USA) and quantified using the ND-2000 (NanoDrop Technologies, Wilmington, NC, USA) (RIN ≥ 6.5, concentrations ≥ 50 ng /μL). An RNA transcriptome library was constructed using a TruSeq™ RNA Sample Prep Kit (Illumina, San Diego, CA, USA). RNA-seq was performed using for paired-end sequencing on an Illumina NovaSeq 6000 sequencing platform, and transcriptome analysis was completed using the Major Bio Cloud online tool [16,17].

### 2.4. Transcriptome Information Analysis

The raw data were base-trimmed and filtered to obtain high-quality cleaned readings. The cleaned reads were aligned to the assembled transcriptome (length ≥ 200 bp) with the Bowtie2 program. The assembled transcripts were annotated into the Rfam database with BLASTX method. Statistical techniques were used to determine the base distribution and quality variations for each cycle of all sequenced reads. Clean reads from the quality control were compared with reference genes to obtain mapped reads for the calculation of transcripts per million (TPM). Differential expression analysis was performed using the R package DESeq2 [18]. The FDR method was used to determine the threshold *p*-value in multiple testing [19], and adjusted *p*-values < 0.05 and |log2(foldchange)| > 1 were set as the thresholds for significant differential expression.

Gene ontology (GO) enrichment analysis and Kyoto Encyclopedia of Genes and Genomes (KEGG) pathway analysis were performed to determine the corresponding functional and metabolic processes of differentially expressed genes (DEGs) using Goatools and R (v 4.3.1)software. The KEGG pathway function was corrected to padjust < 0.05, which was considered significantly enriched for DEGs.

### 2.5. Relative Gene Expression Analysis by Using Quantitative Reverse Transcription RT-qPCR

The microarray results were verified using RT-qPCR. All gene-specific primers (Table S1) were designed using Primer Premier (v5) software, and 16S rDNA was used as the internal control gene. RNA was reverse-transcribed using the HiScript II 1st Strand cDNA Synthesis Kit (Vazyma, Nanjing, China), and real-time PCR was performed using S2×qPCR MIX (Vazyma). Relative transcription of genes was calculated according to the 2^−ΔΔCt^ method [20,21].

### 2.6. Data Analysis

All statistical analyses were performed using the IBM SPSS Statistics software (version 26.0). Significance analysis (*t*-test) was used to compare the hydrolase enzyme, antioxidant enzyme activity, and RT-qPCR of ACP81 at differences NaCl concentrations. Microsoft Excel (version 2021), Origin (version 2023), Mega (version 6.0), and R programming language (version 4.1.0) were used for data processing and graphing. Transcriptome data were analyzed using the MajorBio cloud platform (https://cloud.majorbio.com) (access date 10 February 2023).

## 3. Results

### 3.1. Isolation and Identification of Halotolerant Bacteria

The isolated strain ACP81 was Gram-positive with a cream colony color and a rough surface. The ACP81 was identified as *B. subtilis* through 16S rRNA gene sequencing, with 99% sequence identity (Figure S1), and deposited in the General Microbiology Center of the China Microbial Strain Conservation Committee with the conservation number CGMCC NO. 24288. Table S2 shows the physiological and biochemical properties of the ACP81 strain, which was positive for acetylmethyl carbinol (Voges–Proskauer test), sucrose, glucose, gelatin, D-xylose, and nitrate reduction.

**Figure 1:**
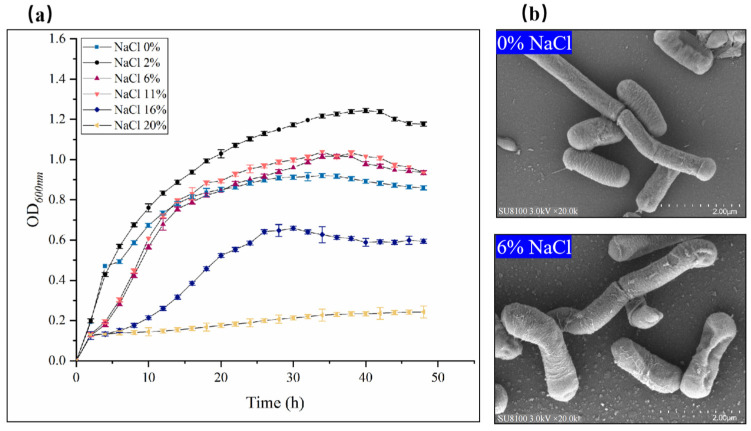
Growth characterization and transmission electron microscopy (TEM) of *B. subtilis* ACP81 under conditions of salt stress. Note: (**a**) Growth of Bacillus subtilis ACP81 strain under salt stress; (**b**) TEM of thin sections of exponential phase cultures grown in LB medium containing 0% or 6% NaCl. Magnification of micrographs is 20,000-fold (white bars below 2.00 μm).

The results show that the growth of the *B. subtilis* ACP81 strain was inhibited with increasing salt concentration, with 2% NaCl and 6% NaCl being the optimal growth concentration and sub-lethal growth concentrations, respectively (Figure 1a). Under 0% NaCl conditions, TEM revealed that the cells were long and rod-shaped with intact cell walls and a slightly wrinkled surface (Figure 1b). In contrast, under sub-lethal salt conditions (6% NaCl), the cells tended toward an oval shape, with evidence of some cell rupture and death (Figure 1b).

### 3.2. Hydrolase Enzymes of B. subtilis ACP81

We found that the *B. subtilis* ACP81 was able to produce cellulase (65.88–72.38 U/mL), α-amylase enzyme (0.23–0.25 U/mL), and neutral protease (3.20–7.13 U/mL) at both 2 and 6% salt concentrations. Cellulase and β-amylase activities significantly increased at the sub-lethal salt concentration, with enzyme activities in the salt groups being 1.10 and 1.61 times higher than the control, respectively, while the activity of neutral protease showed an opposite trend (Table 1, Figure S2).

As shown in Table 2, the superoxide dismutase activity and OFR content of the *B. subtilis* ACP81 decreased considerably at the 6% NaCl concentration by 58.37% (*p* < 0.01) and 43.34% (*p* < 0.05), respectively, compared with the control values (without NaCl). The mean basal activities of POD and CAT were 579.38 and 126.11 nmol/min/g, respectively, and increased at the 6% NaCl concentration by 1.44-fold (*p* < 0.05) and 1.28-fold (*p* < 0.05), respectively.

### 3.3. Genomic Features and Transcriptome Profiles of B. subtilis ACP81

**Figure 2:**
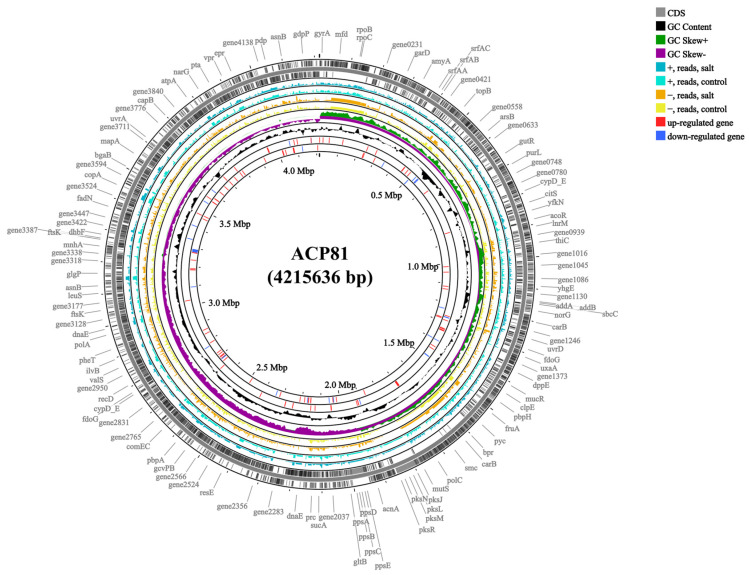
Genome map and gene comparison of *B. subtilis* ACP81. Note: Control indicates 0% NaCl concentration, salt indicates semi-lethal concentration (6% NaCl). First circle: predicted genes; second circle: GC content; third circle: GC skew+: higher than GC average; fourth circle: GC skew−: lower than GC average; fifth circle: read counts mapping to predicted genes on the + strand in 6% NaCl; sixth circle: read counts mapping to predicted genes in 0% NaCl; seventh circle: read counts mapping to predicted genes in 6% NaCl; eighth circle: read counts mapping to predicted genes on the – strand in 0% NaCl; ninth circle: significant DEGs determined based on log2 fold changes (log2 FC) in TPM values that were greater than 2 (upregulated genes, red lines); tenth circle: significant DEGs determined based on log2 fold changes (log2 FC) in TPM values that were smaller than −2 (downregulated genes, blue lines).

Whole-genome sequencing of the *B. subtilis* ACP81 strain yielded a 4,215,636 bp genome with a 43.51% G + C content, 3312 protein-coding genes, 144 CAZyme genes, and 101 predicted RNA genes (Figure 2). We further sequenced the transcriptome of *B. subtilis* ACP81 and created six Illumina libraries using two sets of samples (control and salt groups). On average, 26,048,026 raw RNA reads were obtained for the salt group, with sequence quality scores of 97.12% and 92.18% for Q20 and Q30, respectively (Table S3). Among these samples, from 98.43 to 99.12% of the reads were mapped and used to derive gene expression levels for further analysis of DEGs from TPM reads.

### 3.4. Gene Expression Analysis

**Figure 3:**
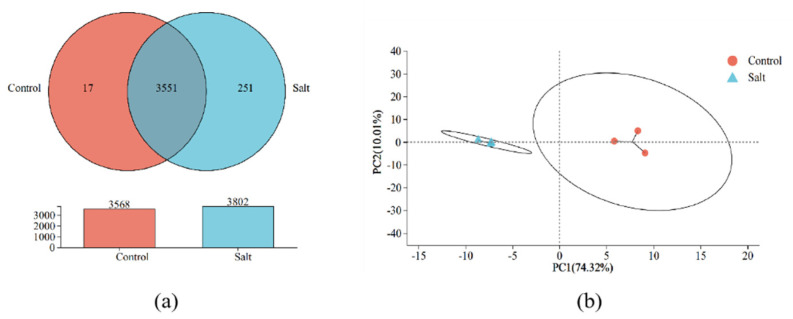
Veen analysis (**a**) and principal component analysis (**b**) of *B. subtilis* ACP81 expression profiles under different levels of salt stress.

Both Venn and principal component analysis results show significant differences in the expression profiles of *B. subtilis* ACP81 under different NaCl stress concentrations. The groups co-expressed a total of 3551 genes; the control specifically expressed 17 genes, whereas the salt group specifically expressed 251 genes (Figure 3a), and the principal component PC1 explained 74.32% of the differences among the six samples (Figure 3b).

**Figure 4:**
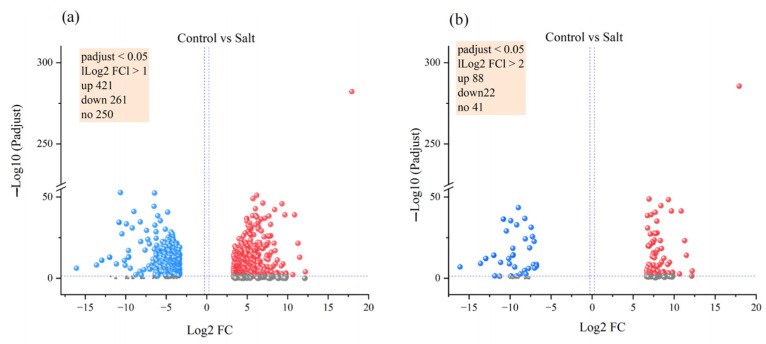
Volcano map of differentially expressed genes between different levels of salt stress. Differentially expressed genes based on the screening criteria of adjust *p* value < 0.05 and |log2 FC| ≥ 1 (**a**) or |log2 FC| ≥ 2 (**b**).

A total of 932 genes were differentially expressed between the control and salt stress groups, including 421 upregulated and 261 downregulated genes (|log2 FC| > 1, padjust < 0.05; Figure 4a). To screen out DEGs more accurately, 82 upregulated genes and 22 downregulated genes were identified (|log2 FC| > 2, padjust < 0.05; Figure 4b, Table S4). The heat map illustrates the top 20 upregulated and downregulated genes in both groups (Figure S3).

### 3.5. GO Functional Enrichment Analysis

**Figure 5:**
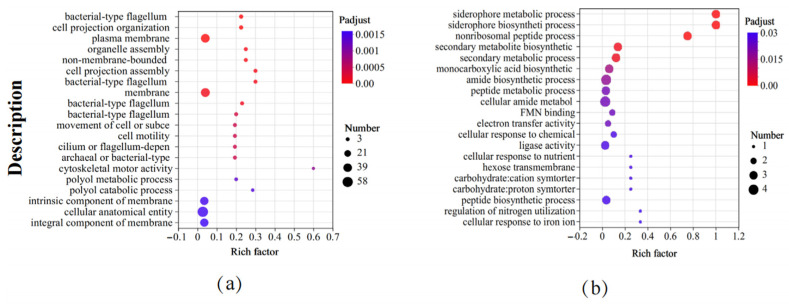
Gene ontology enrichment analysis between different samples. Note: (**a**) The upregulated GO terms in salt stress samples relative to CK samples; (**b**) the downregulated GO terms in salt stress samples relative to CK samples. The X axis represents Rich factor (the ratio of the number of DEGs (sample numbers) enriched in the pathway to the number of all the annotated genes/transcripts (Background number) annotated in the pathway).

We further analyzed the GO functional enrichment of the DEGs in the different groups. Compared to the control, *B. subtilis* ACP81 exposed to salt stress mainly enriched cellular component functions, including the plasma membrane or membrane component, cilium, or flagellum assembly (Figure 5a). Meanwhile, several GO functions of *B. subtilis* ACP81 were decreased under saline stress compared to control conditions, including biological process-like functions, such as secondary metabolite biosynthesis, amide biosynthesis, precipitate metabolic processes, peptide biosynthetic processes, and electron transport. In addition, molecular functions, such as carbohydrate transmembrane transporter activity, ligase activity, and oxidoreductase activity, were lower in the salt group than in the control group (Figure 5b).

### 3.6. KEGG Pathway Enrichment Analysis

**Figure 6:**
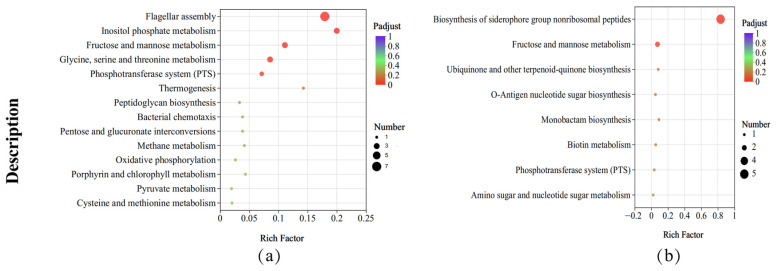
KEGG enrichment analysis between different samples. Note: (**a**) The upregulated KEGG pathways in salt stress samples relative to CK samples; (**b**) the downregulated KEGG pathways in salt stress samples relative to CK samples. The X axis represents Rich factor (the ratio of the number of DEGs (sample numbers) enriched in the pathway to the number of all the annotated genes/transcripts (Background number) annotated in the pathway).

KEGG pathway enrichment analysis revealed that, compared with those of the strains under the control condition, the metabolic pathways of *B. subtilis* ACP81 exposed to salt stress were significantly enriched, such as flagellar assembly (map02040), fructose and mannose metabolism (map00051), cationic antimicrobial peptide resistance (map01503), inositol phosphate metabolism (map00562), PTS (map02060), glycine, serine and threonine metabolism (map00260, map00190) (Figure 6a); in contrast, the biosynthesis of siderophore group non-ribosomal peptides (map01053), O-antigen nucleotide sugar biosynthesis (map00541), monobactam biosynthesis (map00261) and biotin metabolism (map00780) were significantly decreased (Figure 6b). Other molecular processes, including ABC transporters and glycerol lipid metabolism, were significantly altered by salt stress (Table S5).

Seven genes in *B. subtilis* ACP81 involved in the flagellar assembly pathway and five genes involved in the fructose and mannose metabolic pathways were substantially altered in expression (Figure S4) according to the top 20 results of the KEGG enrichment analysis (padjust < 0.05). Therefore, the resistance of *B. subtilis* ACP81 to salt stress involves a complex mechanism comprising numerous metabolic pathways, including flagellar motility, amino acid metabolism, compatible solutes, cationic antimicrobial peptide resistance, and the PTS.

### 3.7. Analysis of Metabolic Pathways Analysis Related to Hydrolytic Enzymes

Transcriptome analysis of genes significantly associated with hydrolases was performed as described in Table S6. The number of genes associated with cellulases and amylases was seven (two upregulated and one downregulated) and eight (six upregulated and one downregulated), respectively. The starch and sucrose metabolism (00500) pathway was enriched in the highest number (three) of all metabolic types (Figure S5). Therefore, oligo-1,6-glucomutase (EC3.2.1.10) and cellobiose PTS permease (EC2.7.1.205) were the key hydrolases produced by the ACP81 strain under salt stress and celC, celB, and malL were the key genes involved in their regulation.

### 3.8. Experimental Validation by RT-qPCR

**Figure 7:**
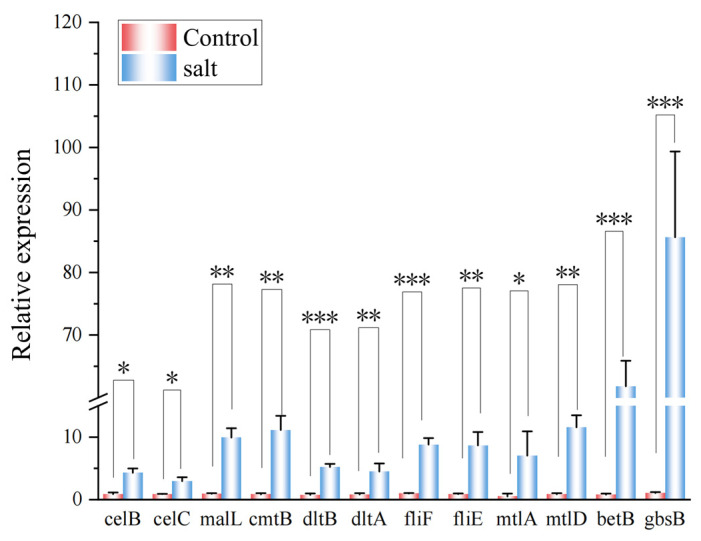
RT-qPCR reveals the upregulation of *B. subtilis* ACP81. Note: *, **, and *** indicate significant differences from the 0% treatment group at *p* < 0.05, *p* < 0.01, and *p* < 0.001, respectively.

RT-qPCR analysis was performed to assess the expression of a range of genes involved in salt tolerance in the *B. subtilis* ACP81. It was found that the expression of genes increased in the salt group, especially the betB and gbsB genes, both of which were 76 and 81 times higher than in the 0% NaCl group, respectively (Figure 7).

To obtain more detail on the role of β-amylase and cellulase-related genes in the regulation of the starch and sugar metabolism pathways, three genes related to β-amylase and cellulase were analyzed using real-time quantitative PCR (RT-qPCR). The main cellulase genes celC and celB encoded by *B. subtilis* ACP81 were upregulated three- and five-fold, respectively, at the 6% salt concentration. The β-amylase gene malL was upregulated ten-fold at the 6% salt concentration compared with the 0% NaCl group. Hence, our results confirm that celC, celB, and malL are core regulatory factors that can positively regulate the expression of cellulase and amylase genes in *B. subtilis.*

## 4. Discussion

### 4.1. Growth and Hydrolase Activities of B. subtilis ACP81 in Response to Salt Stress

Based on studies on the physiological and biochemical properties of bacteria under salt stress, we found that *B. subtilis* ACP81 is a moderately salt-tolerant bacterium and that it exhibits visible changes in cell structure and hydrolytic enzyme activity [22,23].

The results of TEM analysis show that the cell length became smaller and the cell diameter increased when *B. subtilis* ACP81 was exposed to 6% NaCl. Similarly, Wu et al. [24] investigated the mechanism of *B. cereus* JYZ-SD2 resistance to salt ion toxicity and reported the formation of biological periplasm and alteration of cell morphology. Altered cell structure may be responsible for the ability of bacteria to grow in saline environments.

At a sub-lethal concentration (6% NaCl), *B. subtilis* ACP81 showed a significant increase in cellulase and β-amylase activity, but no significant change in α-amylase. This finding is consistent with those of Fouda et al. [25] and Mehmood et al. [26], who reported that extracellular enzymes were produced by *B. cereus* PI-8, *B. subtilis* PI-10, and *B. subtilis* M32 that were found in plant leaves and soil.

β-amylase and cellulases are involved in the starch and sucrose metabolism pathway, which produces maltose that is rapidly converted into glucose [27]. Glucose is a crucial soluble carbohydrate with osmoprotective and compatibility properties under stress situations [28]. Moreover, it has been demonstrated that bacteria can generate extracellular enzymes like amylase and protease, which can attack the fungal cell wall and successfully ward off pathogenic fungi [29,30]. As a result, the ACP81 strain enhances hydrolytic enzyme activity to resist salt stress, which in turn accumulates soluble sugars as a protective mechanism.

The production of antioxidant enzymes is a biochemical means by which organisms respond to metal ion toxicity, which effectively prevents oxidative stress damage to cells [31]. SOD is the main line of defense to scavenge OFRs, rapidly catalyzing O_2_^−^ to O_2_ and H_2_O_2_ [32]; CAT and POD can reduce and reoxidize excess H_2_O_2_, thereby protecting biomolecules from reactive oxygen species-mediated damage [33]. In this study, CAT and POD activities increased significantly at 6% NaCl, 1.28- and 1.44-fold, respectively, compared to activities in 0% NaCl-treated cells (Table 2). These results are confirmatory of those of Li et al. [34] and Ali et al. [35], who reported that *Klebsiella* spp. TN-10 and *Enterobacter* spp. PM23 responded to NaCl shock by producing antioxidative enzymes. Meanwhile, our results also found that OFR content decreased (*p* < 0.01) considerably with the increase in NaCl concentration (*p* < 0.05; Table 2). This suggests that the antioxidant enzymes produced by the salt-tolerant ACP81 strain can scavenge superoxide anion radicals caused by high salt stress, reducing membrane damage and oxidative stress.

### 4.2. Flagellar Assembly and Membrane Plays a Defensive Regulatory Role in ACP81

In this study, we investigated the phenotype and transcriptome responses of ACP81 under sub-lethal salt concentrations and found that seven genes associated with flagellar assembly were upregulated, and the expression of these genes was five to ten-fold higher than that of the control group. This is consistent with the findings of Rajkovic et al. [36], who reported that *B. subtilis* possesses a peptide motif for 5-aminopentanol elongation factor P (EF-P), which is encoded by the flagellar gene. The efficient translation of EF-P can modulate the synthesis of specific diallyl motifs present in proteins required for swarming motility and may also enhance secretory mechanotaxis and flagellin, thereby promoting enhanced motility [37]. In addition, bacteria recover by providing more nutrients and energy to the injured cells via flagellar motility [38]. Nevertheless, the results of a small number of previous studies differ from ours. For example, Cui et al. [38] found that flagellar genes were downregulated, which was attributed to the presence of different EF-P modification pathways in different bacteria.

The cell wall and membrane are the primary defense mechanisms by which bacteria adapt to the external environment. Observations by TEM at the sub-lethal salt concentration revealed that the cell wall of *B. subtilis* ACP81 cells had separated from the cytoplasmic membrane, indicating that it had been lysed (Figure 1b). Transcriptomic data revealed that the expression of dltA, dltB, and dltC increased by more than six-fold at sub-lethal salt concentrations. Woods et al. [39] demonstrated that manipulators of dltA, dltB, and dltC can enhance membrane integrity through the propionylation of D-alanine, which increases the positive surface charge on cell wall phosphopeptides [40,41]. The dltABC gene encodes various ligases and proteins associated with the D-alanine reaction and various naturally occurring cationic antimicrobial peptides [42,43], which have a well-defined α−helix or β−strand secondary structure and display a predominantly +2 to +9 net positive charge [44,45]. This contributes to the preferential aggregation of peptides and proteins on the bacterial surface and enhances membrane density.

Furthermore, the cell walls of Gram-positive bacteria mainly consist of peptidoglycan and phosphopeptidic acid. The secondary structure of lipoteichoic acids (LTAs) is highly dependent on the presence of environmental cations [37], whereas the D-alanylation of LTAs and electrostatic interactions with metal cations can strongly improve the cell wall’s mechanical properties [46]. Studies have shown that the D-alanylation of LTAs increases the density of the cell walls of Gram-positive bacteria, thereby reducing the concentration of effective peptides in the membrane. Consistent with our study, we found that the *B. subtilis* ACP81 harbored an M23 family protein gene, which was upregulated six-fold under sub-lethal salt conditions. The M23-peptidase family performs (D,D)-carboxypeptidation and regulates cell shape [47]. We suggest that the resistance of *B. subtilis* ACP81 to salt stress can be attributed to the presence of flagellin and the biosynthetic regulation of cellular structure through D-alanine propionylation.

### 4.3. Compatible Solutes Play an Osmoregulatory Role in B. subtilis ACP81

Accumulation of compatible substances (low molecular weight amino acids and their derivatives, sugars, or sugar alcohols) was one of the main mechanisms by which cells cope with high osmotic pressure, and their ability to cope depends on the methylation length of the hydrocarbon chain [48,49]. Our transcriptome results show that gbsB (99-fold) and betB (57-fold) were upregulated under salt stress. This analysis is similar to that observed by Oshone et al. [50] and Tang et al. [51] according to which *Frankia* and *Pseudomonas* may be closely related to genes involved in threonine and glutamate synthesis. It has been shown that the gbsB and betB genes encode choline dehydrogenase and glycine betaine aldehyde dehydrogenase, respectively, both of which are involved in glycine betaine synthesis, linked to NADPH [51,52]. In addition, we detected an upregulation in the expression of the serA gene that encodes a stress response protein, 3-phosphoglycerate dehydrogenase, which catalyzes the initial phase of L-serine synthesis [53]. We suggest that the resistance of *B. subtilis* ACP81 to salt stress can be attributed to the biosynthetic regulation of cellular osmosis by glycine, serine, threonine, and choline–glycine betaine.

Transcriptomic data confirmed that the polysaccharide metabolism in the *B. subtilis* ACP81 related to fructose and mannose was significantly altered in response to salt stress, particularly the expression of the mtlD gene (nine-fold), which encodes mannitol-1-phosphate 5-dehydrogenase. This is consistent with the results of Arias et al. [54], who indicated that, the higher the mannose content, the better the salt tolerance of the cells. In addition, salt-tolerant *B. subtilis* HS-5 and LR-1 were obtained from bacon and seawater and fermented in high-salt media to produce the polysaccharide BH-1, the main components of which are mannose and glucose [23]. Extracellular polysaccharide metabolism is accelerated in extreme environments, which helps the strain access water and concentrate nutrients for survival. Moreover, the regulatory process promotes the mechanical stability of the cell membrane and prevents cell death due to dehydration [55]. Therefore, the synthesis of extracellular polysaccharides is a strategy used by microorganisms to adapt to extreme environments.

We also found that cmtB and mtlA were significantly upregulated, and that they were involved in the transport of the PTS sugar transporter subunit IIA, PTS mannitol transporter subunit IICB, and PTS mannose transporter subunit IIABC. Zhang et al. [56] indicated that the PTS is a group of enzymes that transfer the phosphate portion of phosphoenolpyruvate from one part of the system to another through relatively fixed sequences [57]. The phosphoenolpyruvate of bacteria is a key factor in the regulation of carbohydrate metabolism based on the fact that it is primarily involved in the transport and phosphorylation of sugar in the cells [58].

### 4.4. Analysis of Metabolic Pathway Analyses Related to the Hydrolytic Enzyme of ACP81

The results of the transcriptome KEGG pathway enrichment analysis show that there was enrichment in carbohydrate-like metabolic pathways with a focus on starch and sucrose metabolism (map00500). This finding is in agreement with the work by Dong et al. [59], who used transcriptome profiling to show that cellulase-producing strains of *Trichoderma. longibrachiatum* are involved in starch and sucrose metabolic pathways, thereby improving their enzyme secretion efficiency.

We also found that cellulase-related genes (celB and celC) were highly expressed and regulated exoglucanases (EC 2.7.1.205), which in turn affected cellulases (Figure S5). In a similar study, four thermostable cellulase genes (celA, celB, celK, and celS) were reported to be clonally transferred into *B. subtilis* for efficient cellulase production [60,61]. B. subtilis ACP81 also has a secondary pathway that is independent of the phosphotransferase system (PTS) and is involved in starch and sugar metabolism pathways (map00500). The malL gene that encodes a-amylase (EC 3.2.1.0) was induced by salt ions, and it hydrolyzes isomaltose and dextrin to D-glucose (Figure S4, Table S6). Sugars (e.g., sucrose and alginate) act as compatible solutes that regulate cellular osmotic pressure. Moreover, such solutes function as stabilizers in the cell, representing an important adaptive mechanism in salt-tolerant bacteria that allows their survival in high-salt environments [62,63]. In addition, sugar is an important energy source and signaling molecule involved in the metabolic activity of microorganisms [64,65]. Therefore, the high expression of β-amylase and cellulase-related genes regulates enzyme activity and indirectly promotes the bacterial stress response to the abiotic stress of high salt.

## 5. Conclusions

The salt-tolerant bacterium B. subtilis ACP81 was obtained from bamboo shoot-processing waste. Salt stress at sub-lethal salt concentrations altered its cell morphology and increased its cellulase and β-amylase activities. Transcriptomic analysis of strain ACP81 indicated that sub-lethal salt concentrations induced not only the upregulation of genes related to cell structure, such as flagella and biofilms, but also genes involved in the production of osmotic substances, PTS, and sugar metabolism. The results reveal that the mechanism of resistance to salt stress in B. subtilis ACP81 was attributed to flagellar motility, alteration in cell structure, and accumulation of compatible substances. B. subtilis ACP81 is involved in starch and sugar metabolism pathways, and cellulase and β-amylase metabolic processes can accumulate soluble sugars, which can effectively resist salt stress. RT-qPCR experiments were performed to confirm the transcriptome sequencing data. This study contributes to our understanding of the relationship between hydrolase activity and salt tolerance in bacteria. These properties are favorable for the production of industrial enzymes and the development of biological soil amendment strategies.

## Figures and Tables

**Table 1:** Hydrolase activity of strain ACP81 at different salt concentrations.

	Cellulases (U/mL)	Neutral Proteases (U/mL)	α-Amylase (U/mL)	β-Amylase (U/mL)
Control	65.88 ± 2.75 b	7.13 ± 0.93 a	0.25 ± 0.01 a	0.72 ± 0.05 b
Salt	72.38 ± 1.10 a	3.20 ± 0.52 b	0.23 ± 0.01 a	1.16 ± 0.23 a

Note: Control indicates 0% NaCl concentration, salt indicates semi-lethal concentration (6% NaCl). The results are expressed as the means ± standard deviations. Values in the same row with different lower-case letters indicate significant differences to the salt concentrations at *p* ≤ 0.05.

**Table 2:** Antioxidative enzymes activity of *B. subtilis* ACP81 at different salt concentrations.

	Superoxide Dismutase (U/g)	Peroxidase (nmol/min/g)	Malondialdehyde (nmol/min/g)	Catalase (nmol/min/g)	Oxygen Free Radical (nmol/g)
Control	96.39 ± 12.27 a	401.86 ± 17.83 b	5.64 ± 0.91 a	98.42 ± 8.44 b	31.17 ± 5.68 a
Salt	40.12 ± 7.11 b	579.38 ± 91.36 a	7.02 ± 0.71 a	126.11 ± 12.38 a	17.66 ± 5.11 b

Note: Control indicates 0% NaCl concentration, salt indicates semi-lethal concentration (6% NaCl). The results are expressed as the means ± standard deviations. Values in the same row with different lower-case letters indicate significant differences to the salt concentrations at *p* < 0.05.

## Data Availability

The sequencing data have been deposited in NCBI under BioProject ID PRJNA1068806.

## Supplementary Materials

The following supporting information can be downloaded at: https://www.mdpi.com/article/10.3390/microorganisms12020285/s1, Figure S1: Phylogenetic tree of ACP81; Figure S2: Hydrolysis enzymes of ACP81 under salt stress; Figure S3: Heatmap of the control vs. salt groups upregulated and downregulated genes; Figure S4: KEGG enrichment analysis of DEGs under the salt stress; Figure S5: Starch and sucrose metabolism pathway of Bacillus subtilis ACP81; Table S1: qPCR Primer design; Table S2: Physiological and biochemical identification characterization of ACP81; Table S3: Summary of RNA-seq results; Table S4: The DEGs of different salt concentrations; Table S5: KEGG annotation analysis of DEGS in different salt concentrations; Table S6: Significantly differentially expressed genes for cellulase and β-amylase.
