# Supplementary material for: Integrated Genomics and Transcriptomics Provide Insights into Salt Stress Response in Bacillus subtilis ACP81 from Moso Bamboo Shoot (Phyllostachys praecox) Processing Waste
Source: Microorganisms. 2024 Jan 29;12(2):285. doi: 10.3390/microorganisms12020285 (PMC10893186; doi:10.3390/microorganisms12020285)
Supplement: Supplementary file 1 [file microorganisms-12-00285-s001.zip › Supplementary material.pdf]

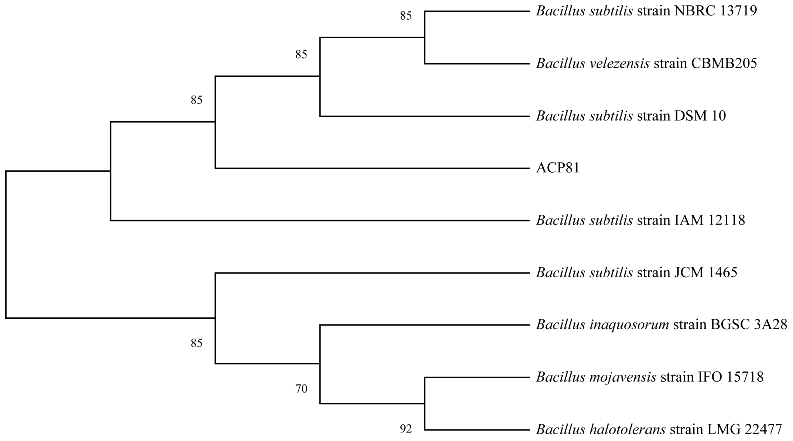

Supplementary Figure S1. Phylogenetic tree of ACP81.

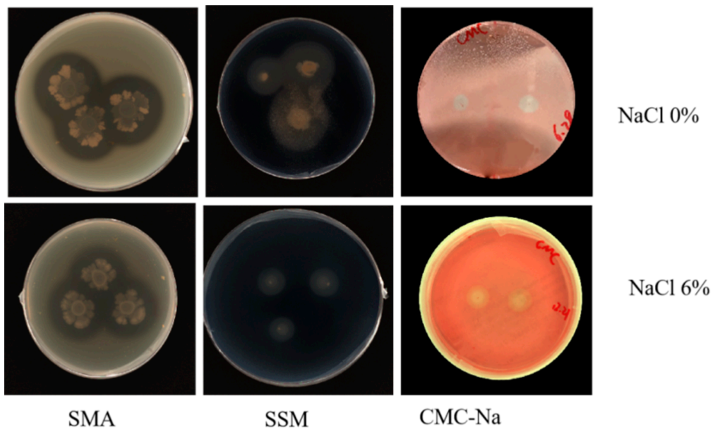

Supplementary Figure S2. Hydrolysis enzymes of ACP81 under salt stress.

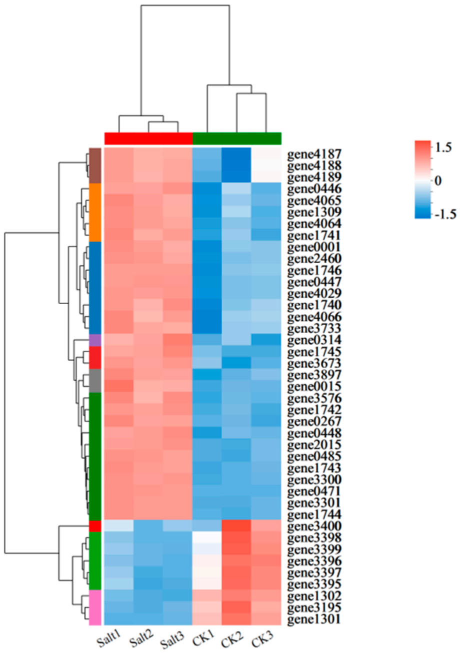

Supplementary Figure S3. Heatmap of the control vs. salt groups upregulated and downregulated genes.

Note: Salt1, Salt2, Salt3 represent the ACP81 culture in medium containing 6% NaCl; N1, N2, N3 represent the ACP81 culture in medium without NaCl.

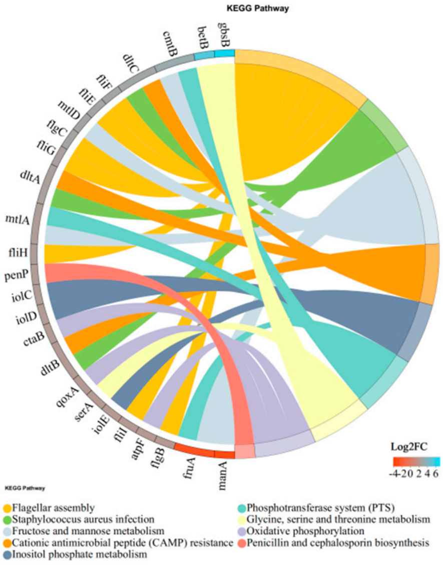

Supplementary Figure S4. KEGG enrichment analysis of DEGs under the salt stress. Note： Gene on the left, KEGG Pathway information on the significant enrichment of differential genes on the right.

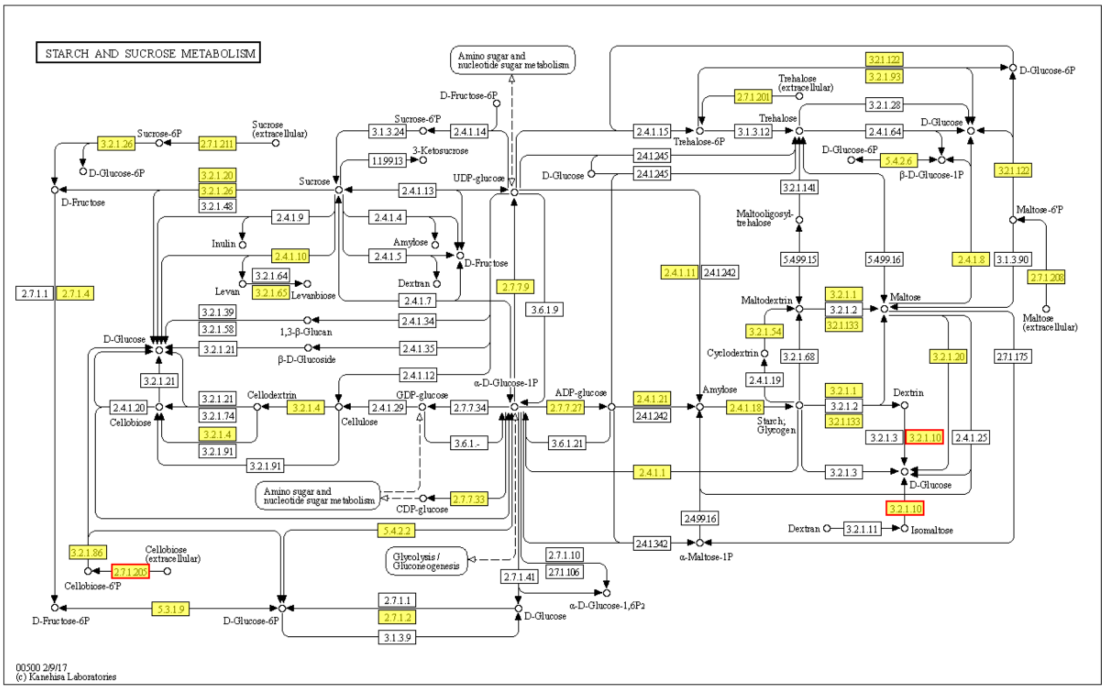

Supplementary Figure S5. Starch and sucrose metabolism pathway of Bacillus subtilis ACP81. Note: the yellow represent the reference genes, the red boxes represent the up-regulated genes.

Supplementary Table S1. qPCR Primer design.

Gene code	Primer name	Sequence design
16S	16SF	CCGTGGAGGGTCATTGGA
	16SR	CGCCTCAGCGTCAGTTGT
betB	betB-F	GGGCAAAACGCATAAAACT
	betB-R	GAAAACCTCTTCCTGAACGA
gbsB	gbsB-F	CGCTTGGCGGTATTATCC
	gbsB-R	TTCGCAGCTTCTTCTGTTG
fliF	fliF-F	TACCGAAAGCGGGAACTAC
	fliF-R	GCTGAACGGAAACAACAATC
fliE	fliE-F	ATTAGTTCGTTAAATGAGTCCCA
	fliE-R	TCTGTTGCTGCAGTTAGAGAGA
mtlA	mtlA-F	TATTGGCGGGTGTATTGGT
	mtlA-R	TTCTTCTGCTTTGGCTTGTT
cmtB	cmtB-F	ATTTCATTGCCATTCCACAC
	cmtB-R	ATGCTCATTATTTTTACCCGC
mtlD	mtlD-F	ATCGGGAGAGGATTTATCG
	mtlD-R	TTTTTTTTCATTGAGGAGGC
dltB	dltB-F	GCGTCTATTCTGCCTTTATTT
	dltB-R	TTCGTCCAAGCCTTCTGTT
dltA	dltA-F	AATGGGTCAAAGAACACGAA
	dltA-R	ACAAATCAAATGAGAACGGC
fruA	fruA-F	TCTTGTCACTGCTCTGTTCGT
	fruA-R	GGCTGCTTTATTTCATTTGC
manA	manA-F	CGGGGGATTTTTTCTATGTG
	manA-R	TGAACTGTATGCCGTTCTGG

Supplementary Table S2. Physiological and biochemical identification characterization of ACP81.

	V-P	Citrate	Propionate	Nitrate reduction	D-xylose	Lactose	Glucose	Sucrose	Gelatine	CMC-Na	pH
Reaction	+	–	+	+	+	–	+	+	+	+	3~9

Note: +positive; –negative.

Supplementary Table S3. Summary of RNA-seq results.

Sample Name	Raw reads	Clean Reads	Q20(%)	Q30(%)	GC content(%)
Z	24,334,783	23,721,700	97.31	92.64	45.61
S	26,048,026	25,392,631	97.12	92.18	45.38

Note: Z indicates NaCl concentration of 0%, S indicates NaCl concentration of 6%. Raw reads: number of double-ended reads of raw sequence data. Clean reads: the number of double-ended reads after quality control; Q20, Q30: the percentage of bases with a Phred value greater than 20 and 30 in the total number of bases after data quality control, respectively.

Supplementary Table S4. The DEGs of different salt concentrations.

Gene_id	Gene name	Log2FC (S/Z)	Pvalue	Padjust	Significant	Regulate	Z	S
gene3300	gbsB	6.206263527	0	0	yes	up	5.47	545.04
gene3301	betB	5.394303676	4.59E-288	9.97E-285	yes	up	4.51	258.15
gene0051	-	2.430663529	2.40E-75	3.47E-72	yes	up	8.94	65.71
gene4196	-	2.08576898	6.47E-67	7.03E-64	yes	up	42.01	242.73
gene0485	poxL	2.227769751	6.27E-66	5.45E-63	yes	up	29.02	185.08
gene2945	-	2.153241955	1.00E-63	7.27E-61	yes	up	8.51	51.36
gene1134	-	2.485848897	7.02E-63	4.36E-60	yes	up	6.48	49.72
gene3897	atpF	2.088331963	4.40E-50	1.59E-47	yes	up	20.92	120.81
gene0448	mtlD	2.797774445	3.51E-49	1.17E-46	yes	up	4.65	43.44
gene4064	dltA	2.525279019	1.57E-45	4.26E-43	yes	up	4.47	34.87
gene1743	fliF	2.907752642	1.89E-42	4.32E-40	yes	up	0.84	8.58
gene0447	cmtB	3.266456203	3.14E-42	6.83E-40	yes	up	4.55	58.63
gene0471	lyxA	2.312895239	1.13E-41	2.05E-39	yes	up	8.67	59.25

gene4029	qoxA	2.161017694	5.56E-40	8.34E-38	yes	up	15.78	94.55
gene0851	-	2.029126348	3.02E-39	4.38E-37	yes	up	21.93	122.93
gene3691	-	2.372903278	6.32E-36	5.97E-34	yes	up	2.06	14.49
gene2553	artQ	2.033787915	7.52E-32	5.45E-30	yes	up	7.35	41.43
gene2015	penP	2.381580268	8.29E-29	5.00E-27	yes	up	2.14	15.12
gene1064	nhaC	2.26393764	1.20E-28	7.17E-27	yes	up	1.35	8.80
gene3795	tagT_U_V	2.33380157	1.99E-28	1.17E-26	yes	up	2.14	14.59
gene3576	opuBD	2.135825648	4.78E-28	2.70E-26	yes	up	6.46	38.68
gene0322	-	2.280035209	2.32E-24	9.99E-23	yes	up	1.77	11.85
gene3133	-	2.502357522	4.58E-24	1.93E-22	yes	up	0.84	6.42
gene3692	-	3.39267519	6.30E-24	2.63E-22	yes	up	0.75	10.83
gene4065	dltB	2.205414465	3.76E-23	1.42E-21	yes	up	2.96	18.49
gene3672	-	2.234982832	1.46E-22	5.42E-21	yes	up	1.68	10.74
gene2460	serA	2.149553608	5.78E-22	1.99E-20	yes	up	1.62	9.58
gene1478	-	2.05183801	8.53E-21	2.63E-19	yes	up	3.31	18.45
gene1036	-	2.087076688	2.32E-20	6.77E-19	yes	up	4.89	28.77
gene1744	fliG	2.545863297	3.35E-19	8.87E-18	yes	up	0.76	6.01
gene0446	mtlA	2.397157453	5.09E-18	1.20E-16	yes	up	5.09	35.75
gene3673	cynT	2.244151676	2.19E-16	4.36E-15	yes	up	2.51	16.51
gene0001	dnaA	2.332516807	5.49E-16	1.03E-14	yes	up	1.17	7.86
gene0267	purT	2.23979063	6.09E-16	1.14E-14	yes	up	0.77	5.02
gene3733	ftsE	2.243228009	3.06E-15	5.30E-14	yes	up	2.83	17.81

gene3389	-	3.456447084	6.59E-15	1.08E-13	yes	up	0.17	2.69
gene0015	dacC	2.205969774	3.29E-14	5.03E-13	yes	up	0.67	4.23
gene1309	ctaB	2.285119734	2.24E-13	3.16E-12	yes	up	1.23	8.08
gene1319	-	2.629831883	3.45E-13	4.75E-12	yes	up	0.82	6.85
gene3307	-	2.550357428	1.03E-10	1.05E-09	yes	up	0.37	2.94
gene1742	fliE	2.838541353	1.37E-10	1.37E-09	yes	up	1.20	11.79
gene1740	flgB	2.069177718	2.53E-09	2.15E-08	yes	up	2.32	12.99
gene1741	flgC	2.772685701	2.92E-09	2.44E-08	yes	up	0.78	7.32
gene3837	-	2.474710647	5.14E-09	4.11E-08	yes	up	0.65	4.68
gene2359	pbuX	2.249829617	6.47E-09	5.02E-08	yes	up	0.33	2.13
gene1746	fliI	2.114216079	6.86E-09	5.28E-08	yes	up	0.48	2.75
gene1745	fliH	2.394257528	2.62E-08	1.88E-07	yes	up	0.48	3.47
gene4323	parA	2.031624409	2.73E-07	1.66E-06	yes	up	0.68	3.71
gene1906	-	2.168458778	3.03E-07	1.82E-06	yes	up	0.73	4.39
gene0259	glpT	2.147708479	2.13E-06	1.11E-05	yes	up	0.26	1.59
gene0314	lip	2.336545755	6.64E-06	3.17E-05	yes	up	0.43	2.97
gene2824	-	2.267388085	9.77E-06	4.56E-05	yes	up	0.25	1.66
gene2970	mreD	2.00258369	1.23E-05	5.62E-05	yes	up	0.79	4.40
gene1748	-	3.670527715	2.51E-05	0.000107406	yes	up	0.12	1.88
gene4143	-	2.417709358	4.61E-05	0.000189309	yes	up	0.86	6.69
gene3351	-	2.61718032	6.61E-05	0.000263171	yes	up	0.29	2.34
gene2233	-	2.089093169	7.25E-05	0.000286319	yes	up	0.54	3.12

gene4066	dltC	2.944393945	0.000152053	0.000566258	yes	up	1.13	11.75
gene4063	-	2.597130135	0.000176259	0.000648622	yes	up	3.82	31.87
gene2017	-	2.205706999	0.000425026	0.001441971	yes	up	1.54	9.57
novel0081	-	2.785714008	0.000511832	0.001703234	yes	up	11.49	117.21
gene3800	capC	2.012643255	0.000565724	0.001866847	yes	up	0.63	3.43
gene1907	-	2.595283109	0.000603849	0.001981373	yes	up	0.19	1.55
gene1375	-	2.292148409	0.000604077	0.001981373	yes	up	0.13	0.87
gene2733	-	2.949784496	0.000618438	0.002023894	yes	up	0.13	1.40
gene4211	-	2.816237014	0.001162045	0.003599605	yes	up	0.27	2.44
gene1493	-	2.218806058	0.001174541	0.003628462	yes	up	0.23	1.42
gene3594	-	2.071991066	0.001289097	0.003945364	yes	up	0.10	0.56
gene2129	-	2.171129952	0.001318378	0.004020822	yes	up	0.50	2.97
gene3880	-	3.204295938	0.002062381	0.005999404	yes	up	0.07	0.98
gene2778	-	2.031068309	0.00277225	0.007838776	yes	up	0.28	1.53
gene1149	-	2.134566721	0.004186371	0.011210086	yes	up	0.09	0.54
gene4188	iolD	2.287326556	0.004365201	0.011638751	yes	up	54.77	349.87
gene2311	nuc	2.373508626	0.005097055	0.013323999	yes	up	0.11	0.81
gene4187	iolE	2.127833792	0.006143358	0.01568686	yes	up	87.32	501.42
gene0263	-	2.686185713	0.006200426	0.015791642	yes	up	0.06	0.55
gene4189	iolC	2.364057149	0.006424906	0.016308674	yes	up	39.50	264.35
gene4186	iolF	2.151654709	0.009271323	0.02251015	yes	up	79.03	459.53
gene2810	-	2.632093353	0.010876551	0.025957985	yes	up	0.15	1.33

gene4136	-	2.159757251	0.012050622	0.028385909	yes	up	0.30	1.73
gene0636	bcr	2.001759917	0.012486921	0.029286648	yes	up	0.09	0.49
gene3390	-	2.171767053	0.01394332	0.032164368	yes	up	1.03	6.74
gene1851	ebrB	2.379879426	0.017283885	0.038719466	yes	up	0.28	1.99

Gene_id	Gene name	Log2FC(S/Z)	Pvalue	Padjust	Significant	Regulate	Z	S
gene1302	manA	-3.20944906	3.00E-56	1.45E-53	yes	down	22.61	3.33
gene1301	fruA	-2.707919953	3.91E-44	9.44E-42	yes	down	19.43	4.01
novel0053	-	-3.257446301	3.08E-37	3.19E-35	yes	down	460.09	69.91
novel0041	-	-3.257446301	3.08E-37	3.19E-35	yes	down	460.09	69.91
novel0029	-	-3.257446301	3.08E-37	3.19E-35	yes	down	460.09	69.91
novel0026	-	-3.257446301	3.08E-37	3.19E-35	yes	down	460.09	69.91
novel0017	-	-3.257446301	3.08E-37	3.19E-35	yes	down	460.09	69.91
novel0003	-	-3.257446301	3.08E-37	3.19E-35	yes	down	460.09	69.91
novel0009	-	-3.257446301	3.08E-37	3.19E-35	yes	down	460.09	69.91
novel0023	-	-3.257446301	3.08E-37	3.19E-35	yes	down	460.09	69.91
novel0019	-	-3.257446301	3.08E-37	3.19E-35	yes	down	460.09	69.91

novel0052	-	-2.986459345	2.73E-36	2.64E-34	yes	down	0.00	0.00
gene0371	-	-2.768104783	1.29E-33	1.06E-31	yes	down	14.87	2.96
gene3654	ykkC	-2.236755285	2.56E-32	1.92E-30	yes	down	38.00	11.05
gene3396	dhbF	-3.149743701	1.43E-29	9.17E-28	yes	down	31.31	4.67
gene3195	bioW	-2.190468753	2.49E-26	1.28E-24	yes	down	48.97	14.52
novel0133	-	-2.453069915	6.35E-25	2.93E-23	yes	down	555.50	145.02
gene1997	exlX	-2.128189835	2.40E-23	9.32E-22	yes	down	51.79	15.88
gene3401	-	-2.292302343	2.57E-19	6.84E-18	yes	down	108.11	29.43
gene3398	entE	-2.907733603	5.03E-19	1.28E-17	yes	down	11.04	1.96
gene1536	-	-2.898843463	5.11E-15	8.67E-14	yes	down	13.57	2.44
novel0008	-	-3.607551278	6.16E-15	1.02E-13	yes	down	1036.43	126.22
novel0016	-	-3.607551278	6.16E-15	1.02E-13	yes	down	1036.43	126.22
novel0022	-	-3.607551278	6.16E-15	1.02E-13	yes	down	1036.43	126.22
novel0040	-	-3.607551278	6.16E-15	1.02E-13	yes	down	1036.43	126.22

novel0025	-	-3.607551278	6.16E-15	1.02E-13	yes	down	1036.43	126.22
novel0028	-	-3.607551278	6.16E-15	1.02E-13	yes	down	1036.43	126.22
gene1537	fldA	-2.908548346	1.03E-14	1.65E-13	yes	down	28.31	5.06
novel0018	-	-3.900634974	6.44E-13	8.63E-12	yes	down	1022.56	97.14
novel0002	-	-3.900634974	6.44E-13	8.63E-12	yes	down	1022.56	97.14
gene1535	fldA	-3.05965201	9.27E-13	1.22E-11	yes	down	12.38	2.02
gene3397	entB	-3.35733212	1.60E-10	1.56E-09	yes	down	45.25	5.88
gene0505	-	-4.095243709	8.37E-10	7.63E-09	yes	down	83.14	6.90
gene3395	mbtH	-3.03276029	1.23E-09	1.09E-08	yes	down	35.49	6.04
gene1442	tnrA	-2.042959579	3.19E-09	2.63E-08	yes	down	29.24	9.57
gene2380	-	-2.153384136	3.70E-09	3.02E-08	yes	down	12.33	3.81
novel0127	-	-2.043457212	3.87E-08	2.68E-07	yes	down	1047.45	370.86
gene0504	-	-4.845829256	1.02E-07	6.65E-07	yes	down	90.85	4.50
gene3399	entC	-2.839992595	1.05E-07	6.85E-07	yes	down	27.69	5.13

novel0180	-	-2.114458305	5.50E-07	3.17E-06	yes	down	0.00	0.00
novel0116	-	-2.330364497	8.37E-07	4.67E-06	yes	down	405.12	118.22
gene4173	-	-2.48394493	1.42E-05	6.40E-05	yes	down	60.90	15.21
gene0553	-	-2.635868405	0.002302912	0.006632948	yes	down	0.35	0.07
gene3400	entA	-2.42619823	0.00275209	0.007791911	yes	down	9.54	2.35

Supplementary Table S5. KEGG annotation analysis of DEGS in different salt concentrations.

	Pathway ID	Pathway Description	Gene Num	Ratio in study	Ratio in pop	P-value	Corrected P-value
up	map02040	Flagellar assembly	7	7/32	39/2554	0	0
	map05150	Staphylococcus aureus infection	3	3/32	5/2554	0	0.0001
	map00051	Fructose and mannose metabolism	5	5/32	27/2554	0	0.0002
	map01503	Cationic antimicrobial peptide (CAMP) resistance	3	3/32	13/2554	0.0005	0.0029
	map00562	Inositol phosphate metabolism	3	3/32	15/2554	0.0007	0.0037
	map02060	Phosphotransferase system (PTS)	3	3/32	28/2554	0.0047	0.0197
	map00260	Glycine, serine and threonine metabolism	3	3/32	35/2554	0.0089	0.0318
	map00190	Oxidative phosphorylation	3	3/32	38/2554	0.0112	0.035
	map00311	Penicillin and cephalosporin biosynthesis	1	1/32	3/2554	0.0371	0.1031

	map02020	Two-component system	4	4/32	135/2554	0.0853	0.1939
	map04714	Thermogenesis	1	1/32	7/2554	0.0846	0.2114
	map00195	Photosynthesis	1	1/32	9/2554	0.1074	0.2238
	map04112	Cell cycle - Caulobacter	1	1/32	12/2554	0.1407	0.2345
	map01501	beta-Lactam resistance	1	1/32	11/2554	0.1297	0.2495
	map00261	Monobactam biosynthesis	1	1/32	11/2554	0.1297	0.2495
	map00910	Nitrogen metabolism	1	1/32	17/2554	0.1935	0.3023
	map00541	O-Antigen nucleotide sugar biosynthesis	1	1/32	20/2554	0.2236	0.3289
	map00680	Methane metabolism	1	1/32	24/2554	0.2621	0.3449
	map00040	Pentose and glucuronate interconversions	1	1/32	26/2554	0.2807	0.3508
	map02030	Bacterial chemotaxis	1	1/32	26/2554	0.2807	0.3508
	map00860	Porphyrin and chlorophyll metabolism	1	1/32	23/2554	0.2527	0.3509
	map02010	ABC transporters	2	2/32	131/2554	0.4949	0.4949
	map00520	Amino sugar and nucleotide sugar metabolism	1	1/32	45/2554	0.4358	0.4952
	map00620	Pyruvate metabolism	1	1/32	51/2554	0.4777	0.4976
	map00270	Cysteine and methionine metabolism	1	1/32	49/2554	0.4641	0.5044
	map01053	Biosynthesis of siderophore group nonribosomal peptides		5/15	6/2554	0	0
	map00051	Fructose and mannose metabolism	2	2/15	27/2554	0.0104	0.0415
	map00130	Ubiquinone and other terpenoid-quinone biosynthesis	1	1/15	12/2554	0.0684	0.1368
down	map00541	O-Antigen nucleotide sugar biosynthesis	1	1/15	20/2554	0.1115	0.1487
	map00261	Monobactam biosynthesis	1	1/15	11/2554	0.0629	0.1676
	map00780	Biotin metabolism	1	1/15	19/2554	0.1062	0.17
	map02060	Phosphotransferase system (PTS)	1	1/15	28/2554	0.1528	0.1746

map00520	Amino sugar and nucleotide sugar metabolism	1	1/15	45/2554	0.2346	0.2346

Supplementary Table S6. Significantly differentially expressed genes for cellulase and β-amylase.

	Gene_id	Gene name	FC(S/Z)	Log2FC(S/Z)	Pvalue	Padjust	Significant	Regulate
cellulase	gene0651	celB	2.651	1.406742886	5.81E-11	6.12E-10	yes	up
	gene0650	celC	2.169	1.116726751	0.022192468	0.048186805	yes	up
	gene4073	celA	1.056	0.078063242	0.69546483	0.793293781	no	up
	gene1227	fabF	1.191	0.252689207	0.028507065	0.059966943	no	up
	gene0361	oxyR	0.812	-0.30120526	0.262003582	0.380062605	no	down
	gene4122	bglS	0.429	-1.220818412	1.15E-14	1.82E-13	yes	down
	gene3044	abfA	0.651	-0.618768194	2.03E-06	1.06E-05	no	down
amylase	gene0328	malL	3.557	1.830501113	1.28E-41	2.23E-39	yes	up
	gene0396	srfAB	3.105	1.634781037	2.52E-29	1.57E-27	yes	up
	gene0395	srfAA	2.688	1.426627644	6.94E-20	1.96E-18	yes	up
	gene0525	sigB	2.098	1.068897774	2.46E-13	3.46E-12	yes	up
	gene0397	srfAC	2.853	1.512715659	3.17E-13	4.40E-12	yes	up
	gene1456	sigI	2.075	1.052802545	1.24E-06	6.71E-06	yes	up
	gene0348	amy A	0.865	-0.20972322268	0.121981693093	0.200568804202	no	up
	gene2803	sigH	0.423	-1.241379751	5.63E-09	4.45E-08	yes	down
